# Epstein-Barr Virus (EBV) Infection in Chinese Children: A Retrospective Study of Age-Specific Prevalence

**DOI:** 10.1371/journal.pone.0099857

**Published:** 2014-06-10

**Authors:** Geng Xiong, Bo Zhang, Mu-yun Huang, Hufeng Zhou, Li-zhen Chen, Qi-sheng Feng, Xi Luo, Hui-jia Lin, Yi-xin Zeng

**Affiliations:** 1 Sun Yat-sen University Cancer Center, State Key Laboratory of Oncology in South China, Collaborative Innovation Center for Cancer Medicine, Guangzhou, P.R. China; 2 Department of Medical Oncology, Cancer Institute and Hospital, Chinese Academy of Medical Sciences/Peking Union Medical College, Beijing, P.R. China; 3 Division of Infectious Diseases, Brigham and Women's Hospital, Boston, Massachusetts, United States of America; 4 Department of Microbiology and Immunobiology, Harvard University, Boston, Massachusetts, United States of America; 5 Department of Otorhinolaryngology, Guangzhou Women and Children's Medical Center, Guangzhou, P.R. China; 6 Neonatal Intense Care Unit, Guangdong Women and Children's Hospital, Guangzhou, P.R. China; Harvard Medical School, United States of America

## Abstract

**Background:**

Epstein-Barr Virus (EBV) is a globally prevalent herpesvirus associated with infectious mononucleosis and many malignancies. The survey on EBV prevalence appears to be important to study EBV-related diseases and determine when to administer prophylactic vaccine. The purpose of this retrospective study was to collect baseline information about the prevalence of EBV infection in Chinese children.

**Methodology/Principal Finding:**

We collected 1778 serum samples from healthy children aged 0 to 10, who were enrolled in conventional health and nutrition examinations without any EBV-related symptom in 2012 and 2013 in North China (n = 973) and South China (n = 805). We detected four EBV-specific antibodies, *i.e.*, anti-VCA-IgG and IgM, anti-EBNA-IgG and anti-EA-IgG, by ELISA, representing all of the phases of EBV infection. The overall EBV seroprevalence in samples from North and South China were 80.78% and 79.38% respectively. The EBV seropositivity rates dropped slightly at age 2, and then increased gradually with age. The seroprevalence became stabilized at over 90% after age 8. In this study, the seroprevalence trends between North and South China showed no difference (P>0.05), and the trends of average antibody concentrations were similar as well (P>0.05).

**Conclusions/Significance:**

EBV seroprevalence became more than 50% before age 3 in Chinese children, and exceed 90% after age 8. This study can be helpful to study the relationship between EBV and EBV-associated diseases, and supportive to EBV vaccine development and implementation.

## Introduction

Epstein-Barr Virus (EBV) was discovered by Epstein, Achong and Barr in 1964, and it was later classified as Human Herpesvirus 4 (HHV4) [1]. EBV is a globally prevalent virus, and over 90% of the world's population are infected with the virus in adulthood [2]. EBV infection begins in the oropharynx and then enters the blood and binds to CD21 receptors on B lymphocytes [3], [4]. EBV infection is associated with many diseases, *e.g.*, infectious mononucleosis (IM), Burkitt's lymphoma, Hodgkin Disease and nasopharyngeal carcinoma (NPC) [5], [6]. Primary acute infection may cause infectious mononucleosis (IM), whereas in most cases it causes asymptomatic seroconversion and the infection becomes latent. It has been demonstrated that latent infection is associated with several malignancies such as Burkitt's lymphoma, Hodgkin Disease, nasopharyngeal carcinoma (NPC) and gastric carcinoma, and thus even latent infection has been a cause of growing concern [7].

In developed countries, people develop the primary infection late in adolescence, particularly after having contact with saliva. Fifty to seventy-four percent of teenagers infected during late adolescence will get infectious mononucleosis, which is also known as the “kissing disease” [8]. However, people in less developed areas experience primary infection much earlier and have a higher morbidity due to EBV-associated malignant diseases than those in developed countries [7]. These differences have led to the proposal of a hypothesis that different periods of EBV infection are related to the severity of the associated diseases [9], [10]. We planned to evaluate EBV prevalence for the future study of EBV-related malignancies like nasopharyngeal carcinoma (NPC) that is prevalent in South China. The morbidity of nasopharygeal carcinoma (NPC) is 30–50/100,000 in South China, but only 3/100,000 in North China. We want to investigate if the EBV prevalence is different between South and North China as well [5].

In addition, HPV prophylactic vaccine has been launched into market for several years. It is suggested that teenager girls are supposed to be immunized before primary infection at the age 10–12 before broad infection [11]. The baseline information of EBV infection in healthy population will be helpful to the development of EBV prophylactic vaccine as well.

## Materials and Methods

### Ethics statement

Approval was obtained from the Sun Yat-sen University Institute Research Ethics Committee. The samples in this study were collected from existing anonymized serum samples from individuals who had undergone conventional health and nutrition examinations previously, so Sun Yat-sen University Institute Research Ethics Committee waived the need of consent.

### Serum samples

Eight hundred and five serum samples from Guangzhou were collected from children between 0 and 10 years old at Guangzhou Women and Children's Medical Center, Guangdong Women and Children's Hospital and the First Affiliated Hospital of Sun Yat-sen University from April to September, 2012. Nine hundred and seventy-three serum samples from Beijing were collected from children between 0 and 10 years old in the Peking Union Medical College Hospital from July, 2012 to March, 2013. All of the children enrolled anonymously in this study were seeking conventional health and nutrition examinations without EBV-related symptoms of rash, fever, lymphadenopathy, pharyngitis, splenomegaly, hepatomegaly, palatal petechiae, or respiratory tract infection symptoms.

The information on the age and gender of the participants is shown in Table 1. Of the children from Guangzhou, 48.9% were male and 51.1% were female. Of the children from Beijing, 53.0% were male and 47.0% were female. The age range was from 1 to 10 years, and the patients were stratified into 10 groups by age (Table 1). Beijing and Guangzhou are both metropolitan cities in North and South China. These cities have many more medical resources than the other cities in the region, so people nearby prefer to seek medical care in these two cities. To some extent, the Beijing and Guangzhou samples are representative of the populations in North and South China, respectively.

**Table 1:** Demographic information for the samples in the study.

Group	Age (years)	Guangzhou	Male/Female	Beijing	Male/Female
**1**	0–1	35	20/15	76	38/38
**2**	1–2	42	20/22	44	22/22
**3**	2–3	60	26/34	53	31/22
**4**	3–4	80	44/36	56	39/17
**5**	4–5	83	45/38	105	54/51
**6**	5–6	94	43/51	103	57/46
**7**	6–7	102	46/56	108	65/43
**8**	7–8	111	55/56	120	65/55
**9**	8–9	103	49/54	172	84/88
**10**	9–10	95	46/49	136	61/75
**Total**		805	394/411	973	516/457

### ELISA analysis

EBV anti-VCA-IgG/IgM, anti-EBNA-IgG and anti-EA-IgG antibodies in the serum were detected according to the manufacturer's protocol provided with the commercial Anti-EBV-CA ELISA (IgG) kit, the Anti-EBV-CA ELISA (IgM) kit, the Anti-EBV-NA ELISA (IgG) kit and the Anti-EBV-EA ELISA (IgG) kit (Euroimmun, Germany). The concentrations of anti-VCA-IgG, anti-EBNA-IgG and anti-EA-IgG were quantified as Relative Unit per milliliter (RU/ml) according to the protocols, and anti-VCA-IgM couldn't be measured in a quantitative way. The overall EBV seroprevalence is defined as anyone of four antibodies shows positive.

### Statistical methods

The statistical significance of the difference in the EBV seroprevalence between Beijing and Guangzhou was determined by the Pearson's Chi-squared test. We also used the Cochran-Mantel-Haenszel Chi-squared test and T test to compare the trends in age-specific EBV seroprevalence and antibody concentrations between Beijing and Guangzhou. R software version 3.0.2 was used for all of the statistical analysis.

## Results

**Figure 1:**
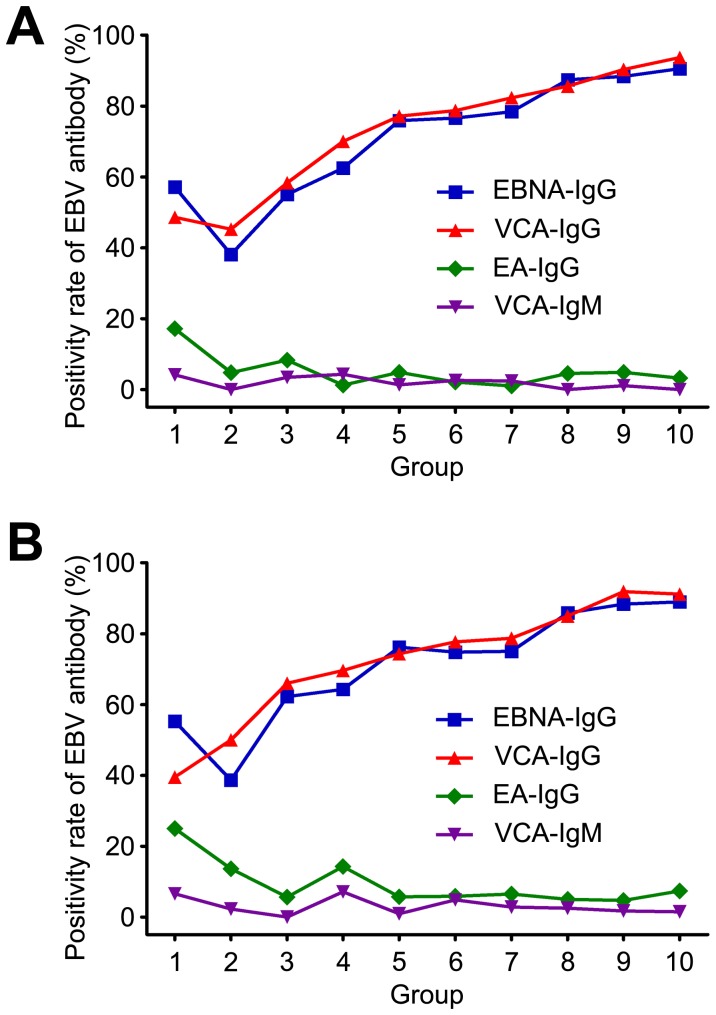
EBV seropositivity rates in Guangzhou and Beijing. (A) Four EBV antibodies were screened in 805 serum samples from Guangzhou. (B) Four EBV antibodies were screened in 973 serum samples from Beijing.

We tested anti-VCA (Viral Capsid Antigen) IgG and IgM antibodies, anti-EA (Early Antigen) IgG and anti-NA (Nuclear Antigen) IgG in all samples that could cover different phases of EBV infection (Table 2) [12]–[16]. Anti-VCA IgG and IgM appear after the initial EBV infection and will rise rapidly during acute infectious mononucleosis [14]. Anti-VCA-IgM disappears after approximately 4 weeks, but anti-VCA-IgG persists for the entire life of the individual. In the Guangzhou samples, the anti-VCA-IgG seropositivity rate was 48.57% at age 0–1, and it declined to 45.24% at age 1–2. It then increased gradually in the older groups and finally peaked at age 8–9, at 90.29% (Figure 1A). In the Beijing counterparts, the anti-VCA-IgG seropositivity rate increased consistently from age 0–1, at 39.47%, and peaked at age 8–9, at 91.86% (Figure 1B). The seropositivity rate for anti-VCA-IgG decreased slightly in Guangzhou at age 1–2. Anti-VCA-IgM was difficult to detect because it declined quickly, and the seropositivity rate was always under 10% in both the Guangzhou and the Beijing samples (Figure 1AB).

**Table 2:** EBV-specific antibody seropositivity status by ELISA.

	Guangzhou					Beijing				
Group	EBNA-IgG(%)	VCA-IgG(%)	EA-IgG(%)	VCA-IgM(%)	Overall(%)	EBNA-IgG(%)	VCA-IgG(%)	EA-IgG(%)	VCA-IgM(%)	Overall(%)
**1**	20/35 (57.14)	17/35 (48.57)	6/35 (17.14)	2/35 (5.71)	20/35 (57.14)	42/76 (55.26)	30/76 (39.47)	19/76 (25.00)	5/76 (6.58)	46/76 (60.53)
**2**	16/42 (38.10)	19/42 (45.24)	2/42 (4.76)	0/42 (0.00)	20/42 (47.62)	17/44 (38.64)	22/44 (50.00)	6/44 (13.64)	1/44 (2.27)	24/44 (54.55)
**3**	33/60 (55.00)	35/60 (58.33)	5/60 (8.33)	2/60 (3.33)	35/60 (58.33)	33/53 (62.26)	35/53 (66.04)	3/53 (5.66)	0/53 (0.00)	36/53 (67.92)
**4**	50/80 (62.50)	56/80 (70.00)	1/80 (1.25)	3/80 (3.75)	56/80 (70.00)	36/56 (64.29)	39/56 (69.64)	8/56 (14.29)	4/56 (7.14)	41/56 (73.21)
**5**	63/83 (75.90)	64/83 (77.11)	4/83 (4.82)	1/83 (1.20)	64/83 (77.11)	80/105 (76.19)	78/105 (74.29)	6/105 (5.71)	1/105 (0.95)	80/105 (76.19)
**6**	72/94 (76.60)	74/94 (78.72)	2/94 (2.13)	2/94 (2.13)	74/94 (78.72)	77/103 (74.76)	80/103 (77.67)	6/103 (5.83)	5/103 (4.85)	83/103 (80.58)
**7**	80/102 (78.43)	84/102 (82.35)	1/102 (0.98)	2/102 (1.96)	88/102 (86.27)	81/108 (75.00)	85/108 (78.70)	7/108 (6.48)	3/108 (2.78)	86/108 (79.63)
**8**	97/111 (87.39)	95/111 (85.59)	5/111 (4.50)	0/111 (0.00)	99/111 (89.19)	103/120 (85.83)	102/120 (85.00)	6/120 (5.00)	3/120 (2.50)	107/120 (89.17)
**9**	91/103 (88.35)	93/103 (90.29)	5/103 (4.85)	1/103 (0.97)	93/103 (90.29)	152/172 (88.37)	158/172 (91.86)	8/172 (4.65)	3/172 (1.74)	158/172 (91.86)
**10**	86/95 (90.53)	89/95 (93.68)	3/95 (3.16)	0/95 (0.00)	90/95 (94.74)	121/136 (88.97)	124/136 (91.18)	10/136 (7.35)	2/136 (1.47)	125/136 (91.91)
**Total**	608/805 (75.53)	626/805 (77.76)	34/805 (4.22)	13/805 (1.61)	639/805 (79.38)	742/973 (76.26)	753/973 (77.39)	79/973 (8.12)	27/973 (2.77)	786/973 (80.78)

Anti-EBNA-IgG appears approximately three months after the initial infection and typically persists for the whole life of the individual, similarly to anti-VCA-IgG; this measurement is typically employed to detect a previous infection [15]. In the Guangzhou samples, the anti-EBNA-IgG seropositivity rate initiated at 57.14% at age 0-1, but it dropped dramatically to 38.10% at age 1–2 and then peaked at age 9–10 at 90.53% (Figure 1A). In the Beijing samples, the anti-EBNA-IgG seropositivity rate initiated at 55.26% at age 0–1, then decreased to 38.64% at age 1–2, and finally peaked at 88.97% at age 9–10 (Figure 1B). The decrease at age 1–2 was because of the disappearance of maternal antibodies, similar to anti-VCA-IgG.

Anti-EA-IgG rises transiently after primary infection because EA is usually expressed in the early phase of lytic replication [17]. Anti-EA-IgG will become undetectable after approximately 3 to 6 months. In the Beijing samples, the anti-EA-IgG positivity rate began at a higher level in 1-year-old children at 25.00%, and it declined to 13.63% at age 1–2. Additionally, the seropositivity rate at age 3–4 was 14.29%, and the rest groups had seropositivity rates under 10% (Figure 1B). The anti-EA-IgG seropositivity rate of the Guangzhou samples was lower than that of the Beijing samples. Only samples from age 0–1 had an anti-EA-IgG seropositivity rate over 10%, at 17.14% (Figure 1A). It is difficult to detect anti-EA-IgG in the serum because the antibodies disappear shortly after the initial infection. Thus, we relied on the seropositivity rates for anti-VCA-IgG and anti-EBNA-IgG to determine the EBV seroprevalence in children. There were only three children in the Beijing samples and four children in the Guangzhou samples who were anti-EA-IgG positive but negative for the other three antibodies, which does not have a dramatic impact on the results.

**Figure 2:**
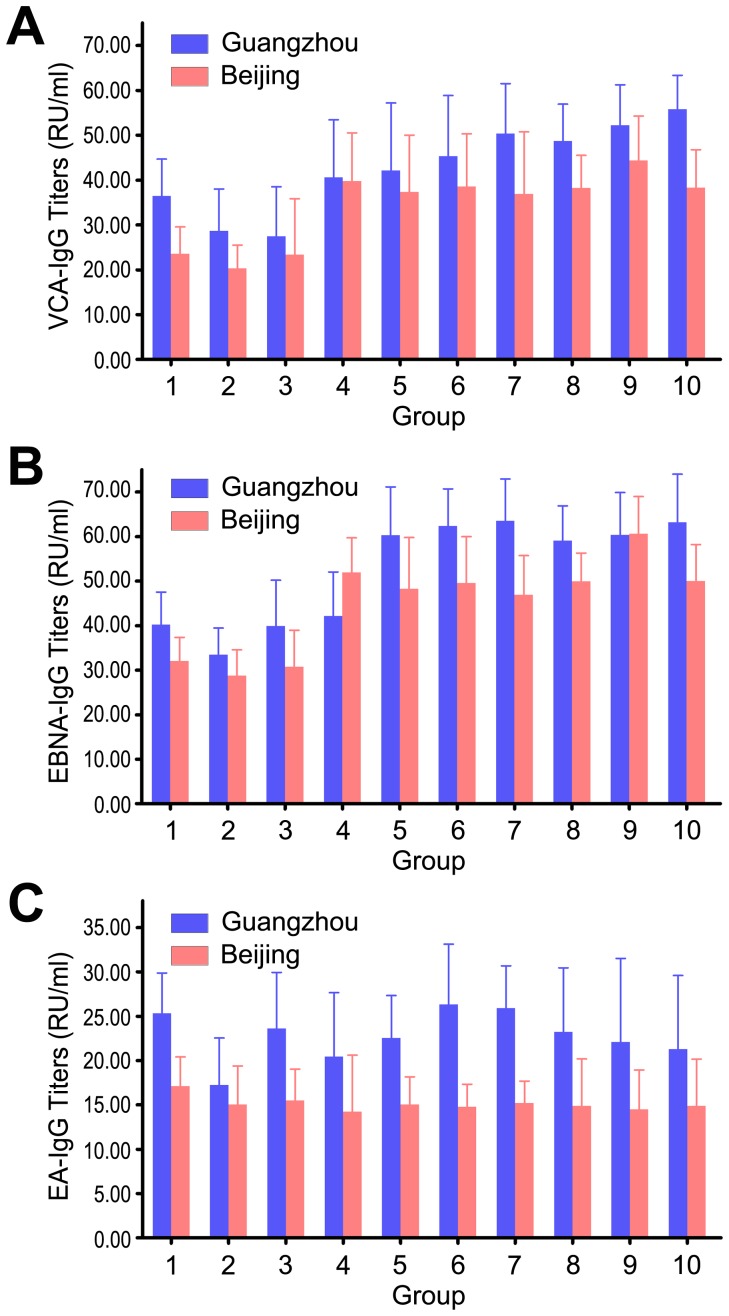
Antibody concentrations of three EBV proteins in Guangzhou and Beijing. (A) Average concentrations of VCA-IgG were measured in all the samples. (B) Average concentrations of EBNA-IgG were measured in all the samples. (C) Average concentrations of EA-IgG were measured in all the samples.

**Figure 3:**
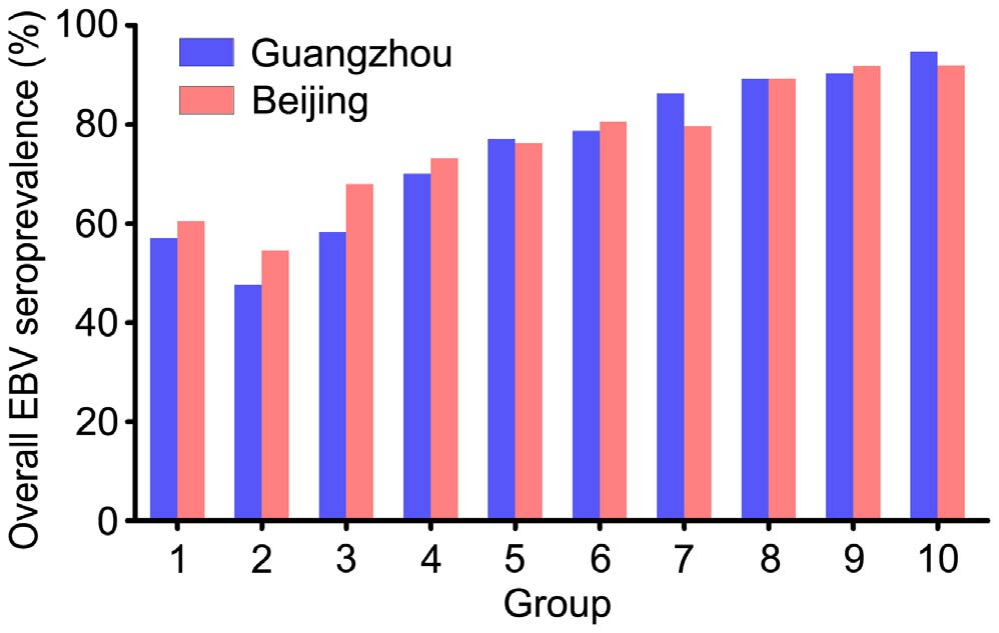
Overall EBV seroprevalence trends in Guangzhou and Beijing.

The EBV seroprevalence in the Beijing was approximately 80.78%, and that of the Guangzhou counterparts was 79.38% (Table 2). The average EBV serological concentrations in the children from Guangzhou were higher than those from Beijing. In the children from Guangzhou, average VCA-IgG concentrations of each group were initiated at 36.43 RU/ml at age 0–1, with drop to 28.67 RU/ml at age 1–2, and then peaked at 55.76 RU/ml at age 9–10. In the counterparts from Beijing, VCA-IgG concentrations also dropped at age 1–2, and peaked at 44.39 RU/ml at age 8–9 (Figure 2A). The trend of EBNA-IgG concentrations was similar to VCA-IgG concentrations, and showed no difference between Guangzhou and Beijing (P>0.05) (Figure 2B). However, the trend of EA-IgG concentrations between Guangzhou and Beijing showed significant difference (P<0.01) (Figure 2C). In summary, the overall EBV seroprevalence in Guangzhou was slightly higher than that in Beijing, but showed no difference (P>0.05) (Figure 3). More than 50% of the children in this study got infected with EBV before age 3, and 90% of them were infected before age 8 (Figure 3).

## Discussion

From two recent studies of EBV seroprevalence in children and teenagers in the U.S. ranging from 6 to 19 years old conducted by the National Health and Nutrition Examination Surveys (NHANES), we know that the seroprevalence of 6 to 8-year-olds during 2009–2010 was 50% and 54.1%, and in 18 and 19-year-olds, the seroprevalence was 82.9 and 89%, respectively [18], [19]. In our study, we targeted children seeking health and nutrition examinations without any EBV infection related symptoms in hospitals. We did not sample from a large population because there is no national health survey in China. However, we made an effort to ensure that the samples in our study would be representative of the population and sufficient in number to show a change in EBV seroprevalence in these areas, similar to the study of non-random samples in England and Wales [20]. Meanwhile, investigators in Wuhan reported their study on EBV serology in 317 Chinese children enrolled in their hospital with suspicion of infectious mononucleosis (IM). They found out that 84% of these Chinese children aged >9 years were infected with EBV [21]. Their study basing on a small group of samples in one hospital could not reflect the landscape of EBV infection in that area, but is complementary to the survey of EBV infection in China.

In our study, the EBV seropositivity rate was higher in newborn babies because maternal antibodies transfer through the placenta [17]. There was a mild decrease in seropositivity in the 2-year-old children as the levels of maternal antibodies declined. The antibody concentrations were not significantly different in the samples from Guangzhou and Beijing. Although EA-IgG concentrations showed significance difference, yet the positivity rate was very low so that we didn't take it into account. In the Beijing samples, the seropositivity rates for the first three groups were slightly higher than their southern counterparts. However, the trend of the seroprevalence distribution stratified by age was not different (P>0.05). This indicated that EBV infection is not geographically variant in China to some extent.

In this retrospective study, the seroprevalence in both Beijing and Guangzhou reached about 90% after age 8. We can tell that more than 80% of Chinese children in these cities were infected with EBV after age 6, and after age 8, 90% became infected. EBV is very easy to spread by saliva contact, and Chinese people had similar baby feeding habits before like mouth feeding or pre-chewing food to feed babies, that resulted in early infection in China. We believe that the popularization of baby formula and changes in baby feeding practice is the major reason of the delay in EBV infection comparing to other developing countries [22]. It takes a lot of efforts to cure patients with EBV related diseases, especially malignancies like NPC and lymphoma. Popularization of baby formula will be more cost-efficient to reduce EBV infection rate and morbidity of EBV related diseases in China. The health practitioners in China should take measures to promote it nationwide.

After the EBV primary infection has been delayed in the infants from Hong Kong and the consumption of salted fish that contain plenty of nitrosamine has been decreased, the NPC incidence in Hong Kong has declined [23]–[25]. This indicates the interplay between the delay of primary EBV infection and lower NPC morbidity in adulthood. In addition, it is also suggested that specific subtypes of Epstein-Barr Virus in Guangdong province, GD1 and GD2, contribute to a higher prevalence of NPC [26], [27]. This implies that not only the period of infection but also the specific subtypes should be considered in our future study of the role that EBV plays in NPC oncogenesis, and we should develop vaccine targeting on GD1 and GD2 subtypes if we try to reduce NPC morbidity. Meanwhile, due to the successful launch of the HPV vaccine into the market, the development of an EBV prophylactic vaccine has drawn more interest [28]. Considering the high prevalence of the virus in the population and the simple route of transmission, the population should be vaccinated before broad infection when EBV prevalence is <50% [19]. It will be controversial to suggest children to receive EBV vaccine before age 3 in China, but we hope EBV prevalence in Chinese population will decrease dramatically in next ten years with socioeconomic development. Meanwhile, EBV vaccine researchers in China should consider more about safety issues and reduce the risk to minimum.
